# Visual insights into translation: demystifying trends of adopting eye-tracking techniques in translation studies

**DOI:** 10.3389/fpsyg.2024.1522168

**Published:** 2024-11-25

**Authors:** Yin Li, Zilong Zhong

**Affiliations:** ^1^Nanyang Institute of Technology, Nanyang, China; ^2^Beijing Foreign Studies University, Beijing, China

**Keywords:** eye-tracking techniques, translation studies, machine translation, human-computer interaction, cognitive effort

## Abstract

**Introduction:**

The increasing use of eye-tracking techniques in translation studies offers valuable insights into cognitive processes and behavioral strategies of translators, reflecting a significant trend within cognitive linguistics and translator training methodologies.

**Methods:**

This review harnesses quantitative bibliometric analysis through Bibliometrix R-package with qualitative content assessment to evaluate the trajectory and thematic evolution of eye-tracking research in translation studies. Through a dataset from the Web of Science, 56 articles were analyzed, revealing distinct thematic dimensions and trend dynamics.

**Results:**

The analysis revealed that eye-tracking is increasingly pivotal in exploring the cognitive and technological dimensions of translation. Central themes include interactions with translation tools, machine translation, and human-computer interaction, highlighting the importance of cognitive research in technology-driven translation. Niche areas such as English-Chinese translation and online consultation suggest specialized topics that warrant further investigation. Additionally, emerging themes like cognitive load and sight translation demonstrate a shift toward exploring real-time translation processing. Declining traditional topics, such as broader translation theories, indicate a growing integration of cognitive research with technological advancements.

**Conclusion:**

These findings elucidate the growth and diversification of eye-tracking applications in translation studies, emphasizing the method’s importance in both academic research and practical applications, thereby informing future studies and enhancing translator training programs.

## 1 Introduction

Eye tracking techniques have emerged as powerful tools for understanding cognitive processes across various domains, including in translation studies (Kornacki, 2019). These techniques, which include both screen-based and mobile eye trackers, measure eye positions and movements, providing valuable insights into how individuals allocate visual attention (Chang, 2009). In the context of translation, eye tracking allows researchers to explore cognitive load and attention distribution, shedding light on the mental strategies employed by translators during the translation process (Płużyczka, 2013). By elucidating these dynamics, eye tracking can significantly enhance translation training methodologies and improve overall translation quality (Hansen, 2013). Previous studies have successfully investigated specific aspects of translation processes adopting eye tracking, such as attention distribution and reading strategies (Gambier and van Doorslaer, 2010). Despite the growing body of research employing eye-tracking techniques in translation studies, a notable gap exists in the form of review within this specialized area. This lack of an overview hampers a holistic understanding of the field’s evolution, research trends, and emerging patterns.

The current review aims to address this gap by providing an analysis of translation studies that utilize eye-tracking techniques, covering the period from 1996 to 2024. By synthesizing findings from multiple studies, this research intends to map out current hotspots and identify emerging trends within the field. Utilizing Bibliometrix, an R-based bibliometric software package (Aria and Cuccurullo, 2017), this study analyzes 56 relevant articles to reveal insights into thematic areas and research trends. This methodological approach allows for providing a clearer understanding of the contributions and development of eye-tracking methodologies in translation research. By highlighting key trends and identifying gaps, the findings are expected to inform both scholars and practitioners in translation studies and cognitive linguistics.

## 2 Data collection

**Figure 1:**
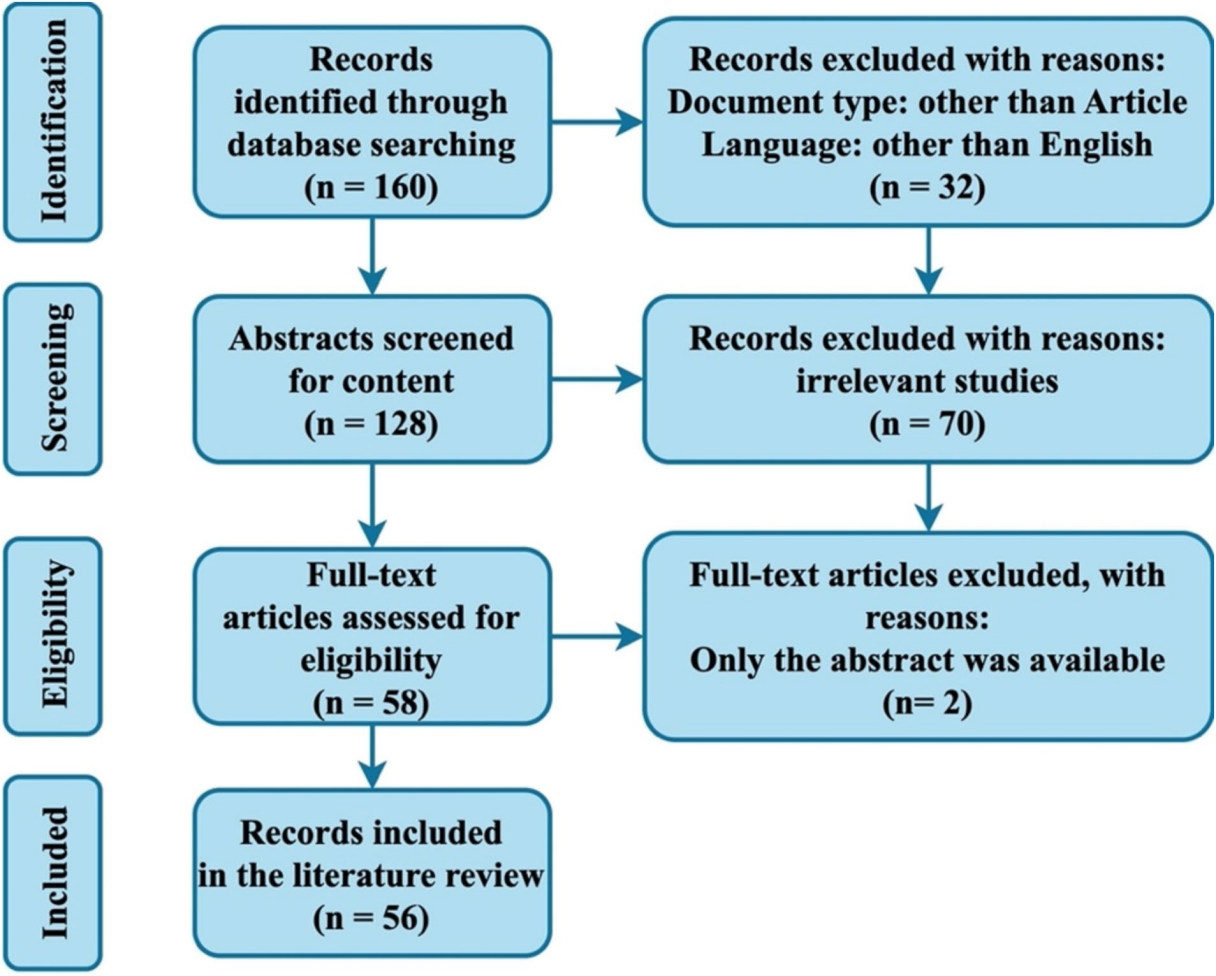
PRISMA flow diagram.

A comprehensive collection of published papers related to translation studies that employed eye-tracking techniques was gathered from the Web of Science (WoS) Core Collection. This collection utilized multiple components of the WoS Core Collection, specifically the Science Citation Index Expanded (SCI-Expanded), Social Sciences Citation Index (SSCI), Arts & Humanities Citation Index (A&HCI), and the Emerging Sources Citation Index (ESCI). The search strategy implemented was: “TI = (‘eye track*’ OR ‘eye movement*’) AND (‘translat*’ OR ‘interpret*’).” Inclusion criteria were clearly defined, as outlined in Figure 1. Data collection occurred on September 30, 2024, resulting in a total of 56 records obtained from 22 journals across 36 WoS categories.

## 3 Results and discussion

### 3.1 Analysis of research themes

**Figure 2:**
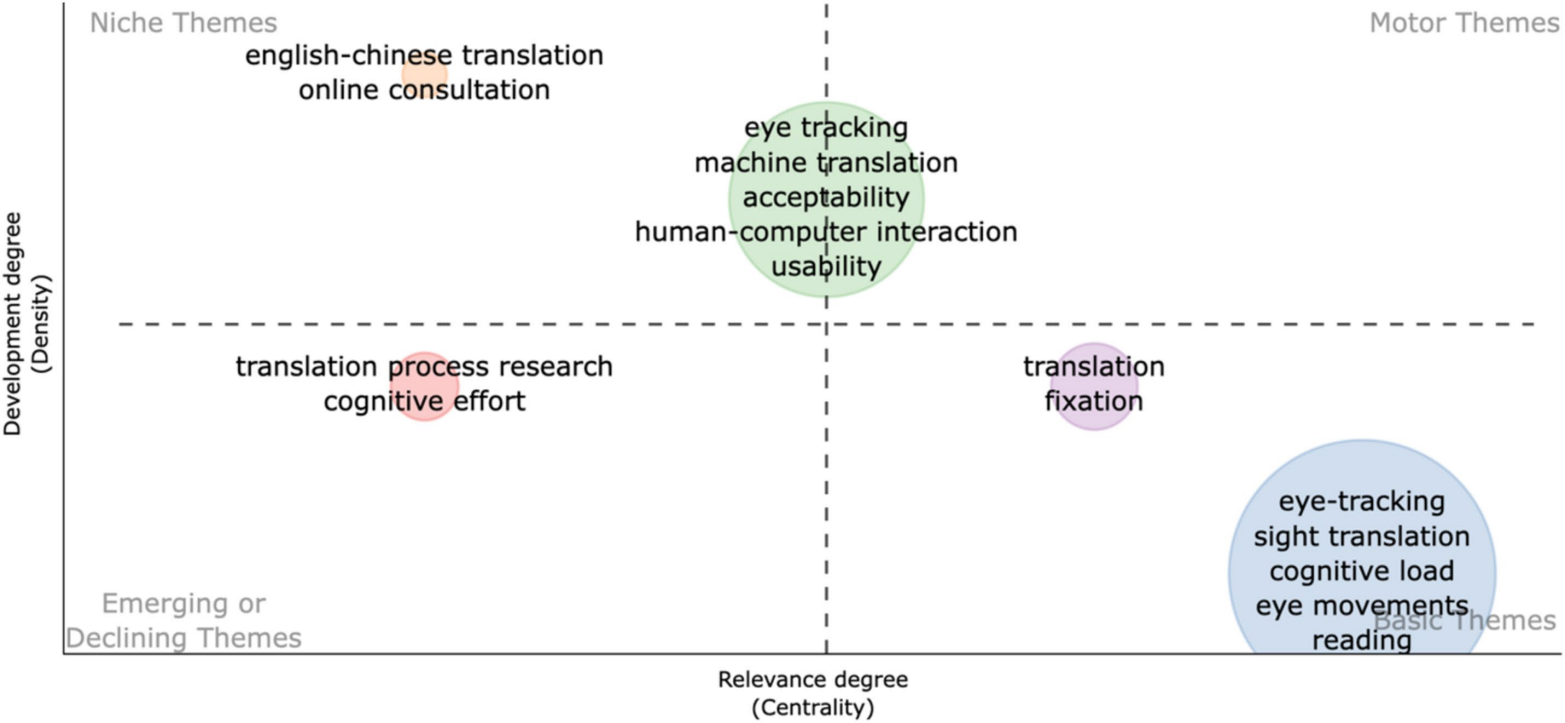
Thematic map of keywords in translation studies employing eye-tracking techniques.

The thematic map of keywords (Figure 2) provides a framework for understanding the dynamics of eye-tracking techniques in translation studies. By utilizing the concepts of density and centrality, we can categorize the identified themes into four distinct quadrants, each representing a unique aspect of the research landscape. This categorization not only aids in identifying core themes but also sheds light on emerging trends and foundational concepts that warrant further exploration.

#### 3.1.1 Motor themes

In the first quadrant, known as the motor themes, we find the most influential topics that exhibit high development and strong connections within the field. Key themes including “eye tracking,” “machine translation,” and “human-computer interaction (HCI)” occupy this space, indicating their pivotal role in advancing the discourse surrounding translation processes. The presence of terms like “acceptability” and “usability” further emphasizes the practical implications of these core themes, highlighting the importance of user-centered approaches in the application of eye-tracking techniques. As a foundational technique in translation studies, “eye tracking” has become a motor theme due to its significant development and integration into various research paradigms (Kruger, 2013). It provides empirical data on cognitive processes during translation, offering insights into how translators interact with text. The robustness of this theme indicates its central role in understanding translation behaviors and strategies. “Machine translation” reflects the intersection of technology and translation practices (Kasperavičienė et al., 2020). With advances in artificial intelligence (AI) and natural language processing, machine translation has garnered attention for its implications on translation quality and efficiency. The strong connections to other themes underscore its relevance in contemporary translation studies, especially as researchers explore user interactions with machine-generated outputs. The prominence of “HCI” in the thematic map highlights the growing recognition of user experience in translation technologies (Doherty and O'Brien, 2014). This theme is crucial for examining how translators engage with digital tools and the cognitive implications of such interactions. As translation increasingly relies on technology, understanding HCI becomes essential for improving usability and acceptability of translation systems. “Acceptability” pertains to the perceived quality and appropriateness of translations, particularly in the context of machine-assisted tools (Cui and Zheng, 2021). Its presence among motor themes indicates a maturing discourse around the criteria by which translations are evaluated. Acceptability connects with cognitive load and usability, emphasizing the need for translators to critically assess the outputs generated by automated systems. Closely related to acceptability, “usability” focuses on the effectiveness, efficiency, and satisfaction with which users can achieve their goals in using translation tools (Doherty and O'Brien, 2014). This theme underscores the importance of user-centered design in translation technologies. A robust understanding of usability can inform the development of eye-tracking studies aimed at improving translator workflows. These motor themes suggest a well-established nexus of research that not only informs current practices but also guides future inquiries into the intricacies of translation.

#### 3.1.2 Niche themes

The second quadrant encapsulates niche themes that, while highly developed, remain somewhat isolated from broader discussions. “English-Chinese translation” and “online consultation” are significant here, indicating specialized areas of focus that demonstrate maturity but may not yet be fully integrated into the larger research framework. “English-Chinese translation” suggests a specialized focus on a particular language pair, reflecting its unique challenges and contexts in translation studies (Cui and Zheng, 2021). Despite its high development, its isolation from broader themes may indicate a need for more integration into the general discourse on translation practices. Researchers could explore cross-linguistic comparisons or the application of eye tracking in understanding specific cultural nuances. “Online consultation” encompasses the dynamics of remote collaboration in translation processes, particularly in the context of professional settings (Cui and Zheng, 2022). Its development suggests that online tools are becoming increasingly significant in translation practice. However, its niche status implies that further exploration is required to understand how online environments impact cognitive processes and decision-making in translation. The isolation of these themes suggests potential opportunities for interdisciplinary collaboration and the exploration of their intersections with broader topics in eye tracking and translation. Researchers might consider investigating how these niche areas can inform and enhance the understanding of user interactions within machine translation systems.

#### 3.1.3 Emerging or declining themes

The third quadrant reveals emerging or declining themes, including “translation process research” and “cognitive effort.” These themes indicate a transitional phase in the research landscape, where certain concepts are gaining attention, while others may be losing relevance. Positioned in the emerging or declining quadrant, “translation process research” reflects an ongoing evolution in understanding how translations are produced (Su and Li, 2019). While it has gained traction, there may be fluctuations in its emphasis within the field. Future research could delve into the cognitive mechanisms involved in real-time translation processes, potentially integrating eye-tracking methodologies to uncover nuanced behaviors. “Cognitive effort” indicates a growing interest in the mental demands placed on translators during the translation process (Schaeffer et al., 2019). As researchers increasingly consider cognitive aspects, this theme’s position suggests that it may be in a transitional phase, possibly overshadowed by more technologically oriented discussions. Exploring the relationship between cognitive effort and eye-tracking data could yield valuable insights into translator performance and efficiency. Researchers should remain vigilant to shifts in this quadrant, as they often signal changing priorities within the field and potential areas for innovation.

#### 3.1.4 Basic themes

The fourth quadrant is characterized by basic themes that, despite being lowly developed, hold the potential to emerge as future research hotspots. Themes such as “eye movements,” “fixation,” “cognitive load,” and “sight translation” represent foundational concepts that are crucial for understanding the mechanisms underlying translation processes. While they may currently lack the robust connections seen in motor themes, their basic nature suggests that they are ripe for exploration and could form the basis for new lines of inquiry. “Eye movements” is foundational for understanding how visual processing impacts translation (Shaikh et al., 2005). Although currently low in development, it presents a rich area for future research. Investigating eye movements in various translation contexts could illuminate patterns that inform both theory and practice. Similar to eye movements, “cognitive load” is a critical concept in translation studies (Ma et al., 2021). Its basic status indicates that while it is recognized, it may not yet be fully exploited in empirical research. Exploring cognitive load through eye-tracking studies could enhance our understanding of translator stress and efficiency. “Sight translation” relates to the instantaneous translation of spoken or written texts without prior preparation (Su and Li, 2019). As a basic theme, it suggests an area ripe for further investigation, particularly in how eye-tracking can reveal cognitive processes involved in sight translation tasks. The theme of “reading” is fundamental to all translation activities. Its basic nature reflects its essential role in the translation process, yet it may require deeper integration with more applied studies. Researching reading patterns through eye-tracking could provide insights into how translators process information in real-time (Lim and Christianson, 2015). As a broad and foundational theme, “translation” represents the core of the field. However, its low development status suggests that there is a need for more specific investigations that can connect traditional translation theories with empirical data gathered through eye-tracking methods. “Fixation” points during reading and translation tasks reveal where attention is directed. As a basic theme, this concept offers a fundamental understanding of visual attention in translation (Kruger, 2013). Investigating fixation patterns can lead to better insights into cognitive strategies employed by translators. Researchers should consider how advancing techniques in eye tracking can illuminate these basic themes and lead to new insights in translation studies.

The findings of the research theme analysis suggest that eye-tracking techniques are poised to play a pivotal role in advancing both the cognitive and technological dimensions of translation studies. The prominence of motor themes, such as eye tracking, machine translation, and HCI, indicates that these areas have become central to the discourse, highlighting the need for continued research on user interactions with translation tools and their cognitive impacts. Meanwhile, niche themes like English-Chinese translation and online consultation point to specialized areas that could benefit from further integration with broader research on translation processes and technology. Emerging and declining themes suggest a shifting focus toward understanding cognitive effort and the translation process, though these areas may require renewed attention to fully explore their potential. Lastly, basic themes related to eye movements, fixation, and cognitive load offer fertile ground for future research, particularly in relation to sight translation and real-time cognitive processing. Overall, these findings underscore the necessity of a more holistic approach that combines cognitive research with advancements in translation technology, paving the way for more effective, user-centered tools and deeper insights into the mental strategies underlying translation tasks.

### 3.2 Analysis of trend topics

**Figure 3:**
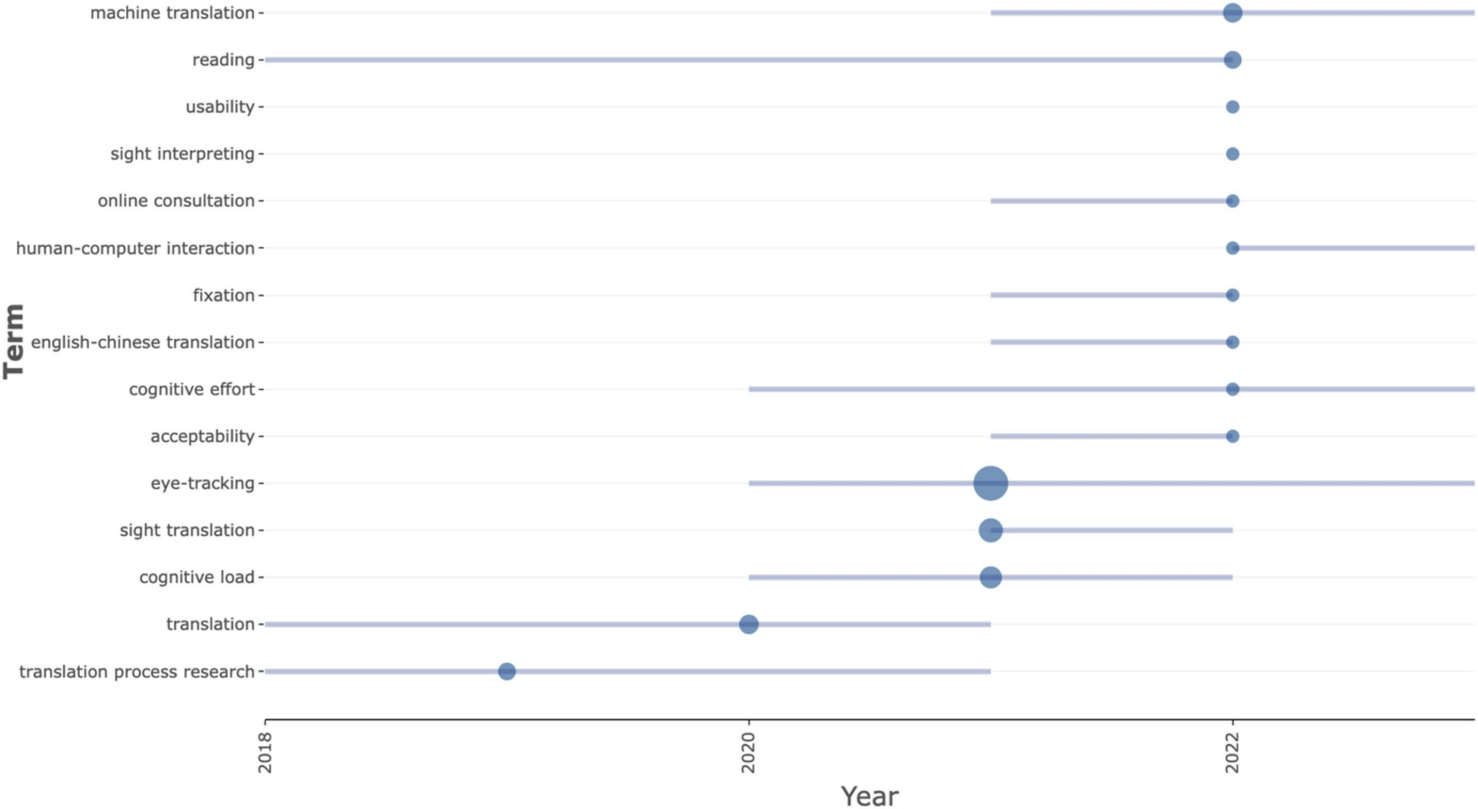
Trend topics by keywords of translation studies adopting eye-tracking techniques.

The exploration of trend topics in translation studies incorporating eye-tracking methodologies illustrates an evolution of research interests and thematic shifts over time (see Figure 3). This analysis provides a comprehensive overview of the changing dynamics within the field, identifying key areas of sustained focus, emerging interests, and potential declines in specific research topics.

#### 3.2.1 Sustained and expanding interest

“Eye-tracking” stands out as the dominant research topic in the field, with significant growth and sustained interest, particularly from 2020 onwards. With a notable presence in 22 publications, the use of eye-tracking techniques in translation studies has gained momentum, reaching its peak median mentions in 2021 and continuing to expand through 2023. This indicates that eye-tracking has evolved from a specialized method to a central tool in translation research, enabling a deeper understanding of cognitive processes, attention distribution, and decision-making strategies during translation tasks.

The sustained focus on eye-tracking highlights both the increasing availability and sophistication of eye-tracking technologies and their growing applicability to various facets of translation. Researchers have leveraged eye-tracking to explore complex translation phenomena such as translator training, workflow optimization, and cognitive effort, which were previously harder to quantify. The expanding scope reflects advancements in the technology itself, such as the development of mobile eye-tracking devices and more powerful data analysis techniques, allowing for more nuanced and sophisticated studies that address real-world translation scenarios. As translation practices continue to evolve, particularly with the advent of machine translation and other assistive technologies, eye-tracking might become one of the key tools in examining the cognitive dynamics at play in these contexts.

#### 3.2.2 Niche and specialized topics

Two specialized topics, “sight translation” and “cognitive load,” have attracted significant attention within the broader field of eye-tracking in translation studies. Both have shown a peak in activity around 2021, suggesting a phase of consolidation in research.

Sight translation, which involves translating orally or writing down a text without prior preparation, has become a focal point for exploring the real-time cognitive processes involved in simultaneous translation tasks. This topic has garnered increasing attention, with eight publications dedicated to the subject, reflecting a growing recognition of its importance in both educational and professional settings. Research in this area has yielded significant insights into how translators manage rapid decision-making, prioritize information, and allocate cognitive resources during sight translation tasks. The continued interest in sight translation is indicative of a broader trend toward examining high-pressure translation tasks that require quick thinking and cognitive flexibility. Cognitive load, another critical area within the field, peaked in 2021 as well. Cognitive load research explores the mental effort involved in translation and how it impacts performance and accuracy. The slight decline in its activity after 2021 suggests that foundational theories related to cognitive load are now well-established, and the field is shifting toward more applied or integrated studies. Specifically, researchers are increasingly interested in examining how cognitive load affects real-world translation tasks, particularly in complex or high-stress scenarios. Although the research focus on cognitive load has slightly diminished, its theoretical implications remain crucial in understanding translator efficiency and mental fatigue.

#### 3.2.3 Emerging research frontiers

“Machine translation” and “HCI” are two emerging topics that are gaining traction in the field of translation studies. Both themes highlight the growing intersection between technology and translation, marking a shift toward more digitally integrated research.

Machine translation has emerged as a key area of focus, with a notable increase in publications exploring how automated translation tools influence the translation process. Appearing in four publications, machine translation studies examine how translators interact with and assess machine-generated outputs, as well as the cognitive and psychological effects of relying on these tools. As machine translation systems improve through AI and machine learning, the need to understand how translators engage with these systems becomes increasingly important. Eye-tracking allows researchers to explore real-time user interaction with machine translations, providing insights into how machine-generated translations affect decision-making, cognitive load, and overall translation quality. HCI in translation has also seen a rise, with two publications in recent years. The focus on HCI reflects an increasing interest in understanding how translators interact with translation tools and digital interfaces. As translation technologies become more sophisticated, understanding how users engage with these systems is crucial for optimizing workflows, improving usability, and minimizing cognitive load. Eye-tracking can provide valuable data on how translators navigate translation interfaces, track their gaze patterns, and assess the usability of machine-assisted translation tools. This growing interest in HCI underscores the broader trend toward user-centered design in translation technologies and the need for tools that facilitate the cognitive processes involved in translation.

#### 3.2.4 Consolidation and decline

Some traditional topics, such as “translation process research” and “translation” itself, have shown a peak in research activity in earlier years, followed by a noticeable decline in recent mentions. This shift indicates a broader pivot within the field from foundational process studies to more technologically driven research, which integrates cognitive theories with the tools and methods of modern translation practices.

Translation process research has been a longstanding area of interest, exploring how translators work through texts, manage decision-making, and engage in cognitive processing. However, as more specific and technology-driven research areas (such as eye-tracking in machine translation and HCI) gain momentum, there has been a reduction in the focus on traditional translation process research. The decline suggests that many of the foundational questions about translation processes have been addressed, and future research may focus more on integrating traditional theories with emerging technologies. Similarly, the theme of translation as a broad concept, while central to the field, has seen a decrease in research activity in recent years. This decline likely reflects a saturation of fundamental studies and a shift toward more specialized investigations that incorporate new technologies or interdisciplinary approaches. The decline in the focus on “translation” as a topic suggests that scholars may now be focusing on more specific aspects of the translation process, such as cognitive load, machine translation, and HCI, rather than revisiting broader theoretical discussions.

#### 3.2.5 Recent interest peaks

Several topics, including “reading,” “acceptability,” “English-Chinese translation,” “fixation,” and “online consultation,” have shown a peak in activity around 2022. These peaks suggest that certain research areas are gaining traction due to new methodologies or specific shifts in industry or educational needs.

Reading has long been a foundational component of translation studies, but recent research has started to delve deeper into how reading patterns and eye movements can shed light on the cognitive dynamics of translation. This growing interest reflects a broader trend toward incorporating empirical data into reading processes, especially in real-time translation tasks. Acceptability studies, which focus on the quality and appropriateness of translations, have become increasingly important. This rise may be linked to the need for higher translation quality, especially in professional and public-facing contexts, such as media, localization, and legal translation. Eye-tracking can provide insights into how translators assess acceptability during the translation process and how cognitive load affects their judgment. English-Chinese translation remains a prominent topic, reflecting the particular challenges posed by this language pair and its importance in the global translation market. Research in this area highlights the unique cognitive processes involved in translating between languages with vastly different syntactic, lexical, and cultural structures. Fixation and online consultation have also seen increased interest, with fixation studies focusing on visual attention patterns during translation tasks, and online consultation reflecting the growing demand for remote translation services. The increase in interest in these topics may be linked to the rise of digital platforms and online collaboration tools that are reshaping the translation industry, necessitating further research into how these factors influence cognitive processing and decision-making.

The findings of trend topic analysis suggest a significant shift toward a more integrated, technology-driven approach to translation studies. The sustained focus on eye-tracking, coupled with emerging themes like machine translation and HCI, highlights the growing importance of cognitive research in understanding how translators engage with digital tools. As eye-tracking technologies continue to evolve, they offer valuable insights into real-time decision-making, cognitive load, and the impact of machine-generated translations, which are crucial for improving translation efficiency and quality. The decline in traditional topics such as translation process research and broader concepts of translation suggests that foundational theories are being increasingly integrated with cutting-edge technologies, marking a pivot toward applied, interdisciplinary studies. Moreover, the rise of specialized topics, such as sight translation and cognitive load, points to a more nuanced understanding of the mental and cognitive demands of translation tasks. These trends indicate that future research will likely continue to focus on the intersection of cognition and technology, with an emphasis on user-centered design and the cognitive dynamics involved in real-world translation practices.

## 4 Future directions

Given the dynamic evolution and the distinct trends identified in the analysis of research themes and trend topics, as well as their temporal distribution in translation studies employing eye-tracking techniques, several promising directions for future research can be proposed (see Table 1). These suggestions aim to build on current strengths, address emerging gaps, and anticipate evolving needs within the field. (1) Advanced integration of eye-tracking with machine translation systems. Future studies could explore deeper integrations of eye-tracking technologies with machine translation tools to reveal how translators interact with AI-generated translations. Research could focus on the cognitive load experienced by translators and how it affects translation quality and efficiency, aiming to optimize human-AI collaboration in translation workflows. (2) Real-time eye-tracking in HCI. With the growing interest in HCI, further research could investigate how real-time eye-tracking data can enhance the usability and design of translation software interfaces. Studies could examine how translators navigate different interface designs and how these impact their cognitive processes and translation strategies. (3) Eye-tracking in sight translation. While sight translation shows a recent peak in research activity, further exploration into how eye movements correlate with cognitive strategies during sight translation could provide deeper insights. This could include studies across different language pairs, particularly those involving complex syntactic structures or scripts. (4) Sector-specific online consultation studies. With online consultation emerging as a key area, future research could focus on specialized contexts such as legal, medical, or technical translations. Eye-tracking could be employed to study how consultants handle on-demand translation, and the cognitive efforts involved, potentially improving remote translation services. (5) Longitudinal studies on cognitive effort and fatigue. Given the importance of cognitive effort and emerging concerns about translator fatigue, longitudinal studies using eye-tracking could assess how cognitive load varies over extended periods and under different working conditions. Such research might lead to better management strategies for cognitive resources in professional translation settings. (6) Cross-linguistic studies on fixation and reading patterns. Research could expand into comparative studies of eye movements across different linguistic systems, examining how script type (e.g., alphabetic vs. logographic) affects reading strategies and fixation patterns during translation tasks. This could inform targeted training programs to enhance reading efficiency and translation accuracy. (7) Eye-tracking in translation education. Future studies could assess the effectiveness of eye-tracking as a tool in educational settings, examining how it can be used to teach and assess translation skills. Research could explore how eye-tracking data correlates with student performance and learning outcomes. (8) Acceptability and quality control. As acceptability becomes increasingly critical, especially in high-stakes translations, further research using eye-tracking could explore how different translation choices impact reader perceptions and satisfaction. Such studies could help develop more rigorous quality assessment frameworks for translations.

**Table 1:** Research gaps and future directions.

	Research gaps	Future directions
1	Limited integration of eye-tracking with machine translation systems.	Advanced integration of eye-tracking with machine translation tools to study cognitive load and its effects on translation quality and efficiency.
2	Underexplored use of real-time eye-tracking in HCI for translation software.	Investigate how real-time eye-tracking data can enhance usability and design of translation software interfaces, examining translators’ navigation and cognitive processes.
3	Insufficient research on eye movements during sight translation, especially for complex language pairs.	Explore the correlation between eye movements and cognitive strategies in sight translation, focusing on different language pairs with complex syntactic structures or scripts.
4	Lack of studies on eye-tracking in sector-specific translation contexts (e.g., legal, medical, technical).	Study eye-tracking in specialized translation contexts such as legal, medical, or technical translations, focusing on cognitive efforts in on-demand translation tasks.
5	Limited longitudinal research on cognitive effort and translator fatigue.	Conduct longitudinal studies using eye-tracking to assess cognitive load over extended periods and under various conditions, aiming to develop better fatigue management strategies.
6	Scarcity of cross-linguistic studies on eye movements and reading patterns.	Conduct cross-linguistic studies examining how script types (e.g., alphabetic vs. logographic) affect eye movements, reading strategies, and fixation patterns during translation tasks.
7	Lack of research on the use of eye-tracking in translation education.	Assess the effectiveness of eye-tracking in teaching and assessing translation skills, exploring its correlation with student performance and learning outcomes.
8	Need for more studies on acceptability and quality control in translation.	Use eye-tracking to investigate how different translation choices affect reader perceptions and satisfaction, contributing to the development of quality control frameworks.

## 5 Conclusion

This review has charted the evolution and integration of eye-tracking techniques in translation studies, highlighting significant strides and identifying fertile areas for future research. The analysis confirms the application of eye-tracking across a spectrum of themes, from the well-established, such as “eye tracking” and “machine translation,” to more isolated yet mature topics like “English-Chinese translation.” It is evident that eye-tracking technology has not only enriched our understanding of the cognitive and linguistic processes in translation but also influenced translation training and practice. The thematic maps and trend analyses point to a dynamic field where traditional processes are increasingly interfaced with cutting-edge technology, underlining the potential for eye-tracking to enhance the efficacy and quality of translation outputs. Furthermore, the emergence of niche and specialized themes suggests a gradual but noticeable shift toward addressing specific linguistic pairings and settings, indicating a maturation within the field that could lead to more targeted and context-specific research. Meanwhile, the rise of themes related to technology use in translation, such as “HCI” and “machine translation,” signals a trend toward digital transformation in the field. These insights not only guide academic research but also impact practical applications, ensuring that translation practices evolve to meet contemporary demands.

In conclusion, the review demonstrates the expansive utility of eye-tracking in translation studies and sets the stage for its continued evolution as a pivotal research tool. By shedding light on both dominant and emergent themes, this review not only reflects the current landscape but also serves as a call to action for researchers to explore underrepresented areas. As the field progresses, it is crucial to leverage these insights to refine translation methodologies and to harness the full potential of technology in translation studies, thereby enhancing both theoretical knowledge and practical applications in this interdisciplinary domain.

## References

[ref1] Aria M. Cuccurullo C. (2017). Bibliometrix: an R-tool for comprehensive science mapping analysis. J. Informet. 11, 959–975. doi: 10.1016/j.joi.2017.08.007

[ref2] Chang C. Y. (2009). Testing applicability of eye-tracking and fMRI to translation and interpreting studies: An investigation into directionality (Doctoral dissertation, Imperial College, London).

[ref3] Cui Y. Zheng B. (2021). Consultation behaviour with online resources in English-Chinese translation: an eye-tracking, screen-recording and retrospective study. Perspectives 29, 740–760. doi: 10.1080/0907676X.2020.1760899

[ref4] Cui Y. Zheng B. (2022). Extralinguistic consultation in English–Chinese translation: a study drawing on eye-tracking and screen-recording data. Front. Psychol. 13:891997. doi: 10.3389/fpsyg.2022.891997, PMID: 35800935 PMC9255674

[ref5] Doherty S. O'Brien S. (2014). Assessing the usability of raw machine translated output: a user-centered study using eye tracking. Int. J. Hum. Comp. Interact. 30, 40–51. doi: 10.1080/10447318.2013.802199

[ref6] Gambier Y. Van Doorslaer L. (2010). Handbook of translation studies, vol. 1. Amsterdam: John Benjamins Publishing Company.

[ref7] Hansen G. (2013). “The translation process as object of research” in The routledge handbook of translation studies. eds. C. Millán and F. Bartrina (Abingdon, England: Routledge), 88–101.

[ref8] Kasperavičienė R. Motiejūnienė J. Patašienė I. (2020). Quality assessment of machine translation output: cognitive evaluation approach in an eye tracking experiment. Texto livre 13, 271–285. doi: 10.35699/1983-3652.2020.24399

[ref9] Kornacki M. (2019). The application of eye-tracking in translator training. Available at: https://www.intralinea.org/specials/article/2421 (Accessed December 20, 2020).

[ref10] Kruger H. (2013). Child and adult readers’ processing of foreignised elements in translated south African picturebooks: an eye-tracking study Target. Int. J. Transl. Stud. 25, 180–227. doi: 10.1075/target.25.2.03kru

[ref11] Lim J. H. Christianson K. (2015). Second language sensitivity to agreement errors: evidence from eye movements during comprehension and translation. Appl. Psycholinguist. 36, 1283–1315. doi: 10.1017/S0142716414000290

[ref12] Ma X. Li D. Hsu Y. Y. (2021). Exploring the impact of word order asymmetry on cognitive load during Chinese–English sight translation: evidence from eye-movement data. Targets 33, 103–131. doi: 10.1075/target.19052.ma

[ref13] Płużyczka M. (2013). “Eye-tracking supported research into sight translation. Lapsological conclusions,’’ in Translation studies and eye-tracking analysis. eds. S. Grucza, M. Pluzycka, and J. Alnajjar (Berlin, Germany: Peter Lang Verlag), 105–138.

[ref14] Schaeffer M. Nitzke J. Tardel A. Oster K. Gutermuth S. Hansen-Schirra S. (2019). Eye-tracking revision processes of translation students and professional translators. Perspectives 27, 589–603. doi: 10.1080/0907676X.2019.1597138

[ref15] Shaikh A. G. Ghasia F. F. Dickman J. D. Angelaki D. E. (2005). Properties of cerebellar fastigial neurons during translation, rotation, and eye movements. J. Neurophysiol. 93, 853–863. doi: 10.1152/jn.00879.2004, PMID: 15371498

[ref16] Su W. Li D. (2019). Identifying translation problems in English-Chinese sight translation: an eye-tracking experiment. Transl. Interpret. Stud. 14, 110–134. doi: 10.1075/tis.00033.su

